# Changes in expression of BDNF and its receptors TrkB and p75NTR in the hippocampus of a dog model of chronic alcoholism and abstinence

**DOI:** 10.1590/1414-431X20154412

**Published:** 2015-06-23

**Authors:** R. Xu, S.R. Duan, J.W. Zhao, C.Y. Wang

**Affiliations:** Neurology Ward of Internal Medicine, First Affiliated Hospital of Harbin Medical University, Harbin, Heilongjiang Province, China

**Keywords:** Chronic alcoholism, Brain-derived neurotrophic factor, Tropomyosin receptor kinase B, p75 Neurotrophin receptor, Immunohistochemistry

## Abstract

Chronic ethanol consumption can produce learning and memory deficits. Brain-derived neurotrophic factor (BDNF) and its receptors affect the pathogenesis of alcoholism. In this study, we examined the expression of BDNF, tropomyosin receptor kinase B (TrkB) and p75 neurotrophin receptor (p75NTR) in the hippocampus of a dog model of chronic alcoholism and abstinence. Twenty domestic dogs (9-10 months old, 15-20 kg; 10 males and 10 females) were obtained from Harbin Medical University. A stable alcoholism model was established through *ad libitum* feeding, and anti-alcohol drug treatment (Zhong Yao Jie Jiu Ling, the main ingredient was the stems of watermelon; developed in our laboratory), at low- and high-doses, was carried out. The Zhong Yao Jie Jiu Ling was effective for the alcoholism in dogs. The morphology of hippocampal neurons was evaluated using hematoxylin-eosin staining. The number and morphological features of BDNF, TrkB and p75NTR-positive neurons in the dentate gyrus (DG), and the CA1, CA3 and CA4 regions of the hippocampus were observed using immunohistochemistry. One-way ANOVA was used to determine differences in BDNF, TrkB and p75NTR expression. BDNF, TrkB and p75NTR-positive cells were mainly localized in the granular cell layer of the DG and in the pyramidal cell layer of the CA1, CA3 and CA4 regions (DG>CA1>CA3>CA4). Expression levels of both BDNF and TrkB were decreased in chronic alcoholism, and increased after abstinence. The CA4 region appeared to show the greatest differences. Changes in p75NTR expression were the opposite of those of BDNF and TrkB, with the greatest differences observed in the DG and CA4 regions.

## Introduction

Chronic ethanol consumption can negatively affect central nervous system function and produce learning and memory impairment in animals and humans (1 2 3). The hippocampus, a region important for memory function, can recover from memory dysfunction caused by prolonged ethanol intake (4 5 6 7). Electrophysiological, morphological and behavioral studies have shown that changes in the hippocampus can be induced by chronic alcohol intake (8,9). For the age group 15-64 years in the European Union, 3.4% of women and 15.3% of men are heavy drinkers, 1.5% of women and 5.4% of men have alcohol dependence, and 1 in 13 deaths in women and 1 in 7 deaths in men are caused by alcohol consumption (10).

Chronic ethanol consumption may play a role in the expression of neurotrophins and their receptors, or interfere with neural signal transduction pathways involving these proteins (11). The neurotrophin family includes neurotrophin-3 (NT-3), neurotrophin 4/5 (NT-4/5), nerve growth factor (NGF) and brain-derived neurotrophic factor (BDNF) (12,13). Neurotrophin signaling is mediated by the low-affinity p75NTR receptor (14). NT-4/5 and BDNF can also bind to the tropomyosin receptor kinase B (TrkB) receptor (15,16). As a neurotrophin associated with the survival and development of neurons, BDNF can regulate the activity of neurotransmitter systems (17,18). Compared with other neurotrophic factors, BDNF may be directly involved in the development and pathogenesis of alcohol dependence (19 20 21 22). It has been reported that the *BDNF* gene locus is associated with alcohol use (23), and even moderate ethanol intake can increase expression levels of BDNF (24). The Val66Met polymorphism in the *BDNF* gene is associated with an earlier occurrence of alcohol dependence and a higher risk of relapse (25). TrkB is activated by BDNF, and its downstream signaling pathways play an important role in suppressing epileptogenesis in the hippocampus (26).

In this study, we examined the expression of BDNF, as well as its receptors TrkB and p75NTR, in chronic alcoholic and abstaining dogs. Our aim was to provide insight into the molecular pathogenetic changes produced by alcohol in the nervous system, in an effort to identify potential therapeutic targets for treating alcohol-related behavioral disorders.

## Animals and Methods

### Animals

Dogs were provided by Professor Chen Huachang of the Psychiatry Department of the First Hospital of Harbin Medical University and Pharmacology Research Base of Harbin Medical University. After 1-2 weeks of adaptation and observation, 20 domestic dogs (9-10 months old, 15-20 kg; 10 males and 10 females) were fed diets mixed with 4 mL/kg alcohol (10, 20, 30, 40 or 50% of the total diet) for 5 days. After feeding the 50% diet, the dose was gradually increased to 8 mL/kg (to produce a drunken state) and maintained for 6 months. Body weights were measured each week. Subsequently, each animal was provided with a 2-kg diet mixed with alcohol and a 2-kg diet without alcohol for 3 days. Dogs that consumed significantly more diet mixed with alcohol than without alcohol (P<0.01) were used as a model of chronic alcoholism.

Chronic alcoholic dogs were divided into alcoholism, low-dose drug intervention (Zhong Yao Jie Jiu Ling, whose main ingredient was the stems of watermelon; developed in our laboratory) and high-dose drug intervention groups, with 5 dogs in each group. The groups were fed saline solution (0.5 mL/kg), drug at 5 mg/kg and drug at 10 mg/kg, respectively, 1 h after dinner by gavage once a day for 7 consecutive days. Subsequently, each animal was provided with 2-kg diet mixed with alcohol and 2-kg diet without alcohol for 3 days. Food intake and body weight were determined. This research protocol was approved by the Animal Experiment Ethics Committee of the First Affiliated Hospital of Harbin Medical University.

### Hematoxylin-eosin staining of neurons in the hippocampus of alcoholic dogs

The animals were intravenously anesthetized by injection of phenobarbital, and bled through the femoral artery. The brains were removed and immersed in 4% formaldehyde for 48 h, then removed and dehydrated in a dehydrator overnight before embedding. Brains were cut into 2.5-μm sections and then baked for 20 min. Subsequently, sections were dewaxed with xylene three times for 10, 5 and 5 min. Then, sections were washed with 100, 95, 90 and 85% ethyl alcohol solutions for 1 min each and washed with water for 2 min. Next, sections were stained with hematoxylin, differentiated with hydrochloric acid alcohol solution, treated with 1% ammonia, and stained with eosin. After washing with water for 1 min, and then 85, 90, 95 and 100% ethyl alcohol for 1 min each, sections were cleared in xylene for 2 min and cover-slipped with resin.

### Immunohistochemistry

Brains were removed, dehydrated and sectioned as described above. Sections were then baked in a 70°C oven for 4 h. Thereafter, sections were dewaxed with xylene three times for 10 min each. After two washes in absolute ethanol for 30 s to 1 min, sections were incubated for 8-10 min in 3% hydrogen peroxide to block endogenous peroxidase, and then washed in a graded ethanol series of decreasing concentration. Sections were subsequently washed with water and distilled water three times, and then incubated in phosphate-buffered saline (PBS). After antigen retrieval and washing with PBS containing Tween, sections were blocked with 50 μL goat serum (Boster, China) for 20 min. Subsequently, sections were incubated with goat anti-BDNF, rabbit anti-TrkB or rabbit anti-p75NTR (ZSGB-Bio, China) antibody at 4°C overnight. After rewarming for 30 min, sections were incubated with 50 μL secondary antibodies PV-9003 Polink-2 plus Polymer HRP Detection System For Goat Primary Antibody and PV-9000 2-step plus¯Poly-HRP Anti-Mouse/Rabbit IgG Detection System (ZSGB-Bio, China) for 20 min. Sections were washed with PBS containing Tween and then incubated with 3,3′-diaminobenzidine tetrahydrochloride (DAB; ZSGB-Bio, China), stained with hematoxylin, differentiated with hydrochloric acid alcohol solution, treated with 1% ammonia, dehydrated with ethanol and dried overnight. Sections were cleared in xylene for 10 min and cover-slipped with resin. The number of cells positive for BDNF, TrkB and p75NTR was assessed in the granular cell layer of the DG and the pyramidal cell layer of the CA1, CA3 and CA4 regions.

### Statistical analysis

Four non-redundant fields (400×) were selected randomly under an optical microscope (Leica, Germany) for each section, and the number of cells positive for BDNF, TrkB and p75NTR were calculated and averaged. Data are reported as means±SD. Statistical analysis was conducted by one-way ANOVA using SPSS Statistics for Windows, Version 17.0 (IBM Corp., USA). P<0.05 was considered to indicate statistical significance.

## Results

### HE staining in the hippocampus of alcoholic dogs

**Figure 1:**
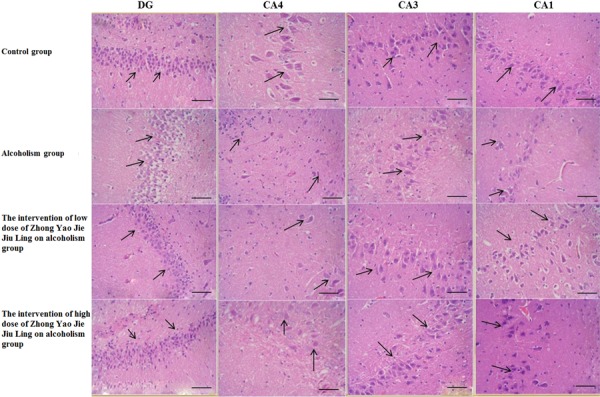
Hematoxylin-eosin (HE) staining of the dentate gyrus (DG) and hippocampus (CA4, CA3, and CA1) in the four groups (bars: 100 mm). The arrows indicate neurons.

In the alcoholism group, hippocampal neurons were reduced in number, and characterized by a diffuse structure, disordered arrangement, absence of a nucleolus, and a hyperchromatic and pyknotic nucleus. Halos surrounded part of the cytoplasm, and some vacuoles showed signs of degeneration, with wider spacing and an irregular arrangement. In the alcoholism drug treatment groups, hippocampal neurons were increased in number and showed an orderly arrangement, and glial cell proliferation was evident. There were no obvious morphological differences between the low- and high-dose drug treatment groups (Figure 1).

### HE staining of neurons in the hippocampus of normal dogs

In the control group, hippocampal neurons exhibited a normal morphology, were numerous, and had an orderly and compact arrangement. They displayed a large nucleoplasmic ratio, a well-defined nucleolus, as well as a circular or ellipsoid nucleus (Figure 1).

### BDNF immunohistochemical staining

**Figure 2:**
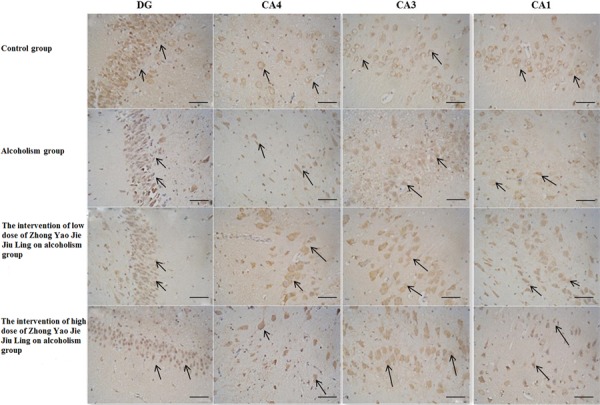
Brain-derived neurotrophic factor (BDNF)-positive neurons in the dentate gyrus (DG) and hippocampus (CA4, CA3, and CA1) in the four groups (bars: 100 mm). The arrows indicate BDNF-positive neurons.

In the hippocampus, BDNF-positive cells were mainly concentrated in the granular cell layer of the DG and in the pyramidal cell layer of the CA1, CA3 and CA4 regions (Figure 2). The number of BDNF-positive neurons in the different regions decreased in the following order: DG, CA1, CA3 and CA4. Compared with the control group, the number of BDNF-positive cells in the granular cell layer of the DG and in the pyramidal cell layer of the CA1, CA3 and CA4 regions were significantly decreased in the alcoholism group (P<0.05). Compared with the alcoholism group, the numbers of BDNF-positive cells in the granular cell layer of the DG and in the pyramidal cell layer of the CA1, CA3 and CA4 regions were significantly increased in the high-dose drug treatment group (P<0.05). Furthermore, compared with the alcoholism group, the numbers of BDNF-positive cells in the granular cell layer of the DG and in the pyramidal cell layer of the CA1, CA3 and CA4 regions were increased in the high-dose drug treatment group, but only the CA4 region showed a statistically significant difference (P<0.05). Moreover, the DG, and the CA1, CA2, CA3 and CA4 regions displayed significant differences between the high-dose drug treatment and control groups (P<0.05). Only the CA4 region in the low-dose drug treatment group showed a statistically significant difference from control (P<0.05; Table 1).

**Table:**
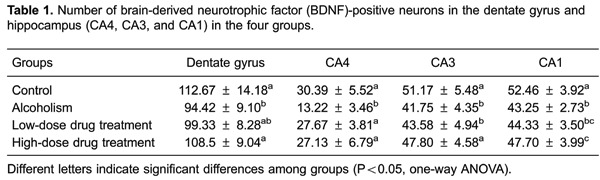

In the control group, pyramidal cells in the CA1, CA3 and CA4 regions were densely arranged, and had longer processes and were deeply stained. In the alcoholism group, pyramidal cells in the CA1, CA3 and CA4 regions were loosely arranged and reduced in number, but in the few remaining BDNF-positive neurons, the cytoplasmic staining was noticeably darker, and the nucleus was clearly visible. In the low- and high-dose drug treatment groups, pyramidal cells in the CA1, CA3 and CA4 regions were densely arranged, but were reduced in number. Granulosa cells in the DG showed similar changes, and changes in their number were more substantial.

### TrkB immunohistochemical staining

**Figure 3:**
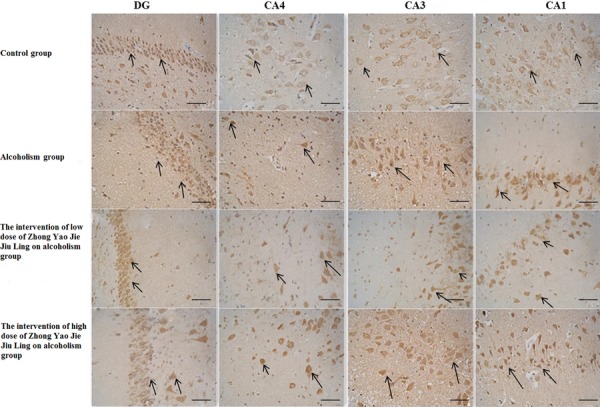
Tropomyosin receptor kinase B (TrkB)-positive neurons in the dentate gyrus (DG) and hippocampus (CA4, CA3, and CA1) in the four groups (bars: 100 mm). The arrows indicate TrkB-positive neurons.

In the hippocampus, TrkB-positive cells were mainly concentrated in the granular cell layer of the DG and in the pyramidal cell layer of the CA1, CA3 and CA4 regions (Figure 3). The number of TrkB-positive neurons in the four regions decreased in the following order: DG, CA1, CA3 and CA4. Compared with the control group, the numbers of TrkB-positive cells in the pyramidal cell layer of the CA1, CA3 and CA4 regions were significantly reduced in the alcoholism group (P<0.05). Compared with the alcoholism group, the numbers of TrkB-positive cells in the DG, and in the CA1, CA3 and CA4 regions were not significantly changed in either the low- or high-dose drug treatment group (P>0.05). Additionally, compared with the alcoholism group, the numbers of TrkB-positive pyramidal cells in the CA1, CA3 and CA4 regions were increased in the low-dose drug treatment group, but only the CA1 and CA4 regions showed a significant difference (P<0.05). Furthermore, the low- and high-dose drug treatment groups showed no significant difference from the control group (P>0.05), although the numbers were not as high (Table 2).

**Table:**
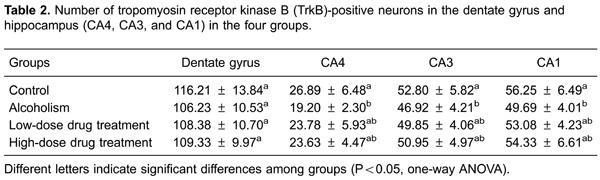

In the control group, pyramidal cells in the CA1, CA3 and CA4 regions were densely arranged, and had long processes and were deeply stained. In the alcoholism group, cells in the CA1, CA3 and CA4 regions showed signs of necrosis, were decreased in number, and were loosely arranged. Furthermore, the pyramidal cell layer was thinner, nuclei appeared light, the extracellular matrix seemed loose, and microcavity formation was evident. In the low- and high-dose drug treatment groups, pyramidal cells in the CA1, CA3 and CA4 regions were densely arranged, but were fewer in number. Granulosa cells in the DG showed similar changes, although changes in their number were more substantial.

### p75NTR immunohistochemical staining

**Figure 4:**
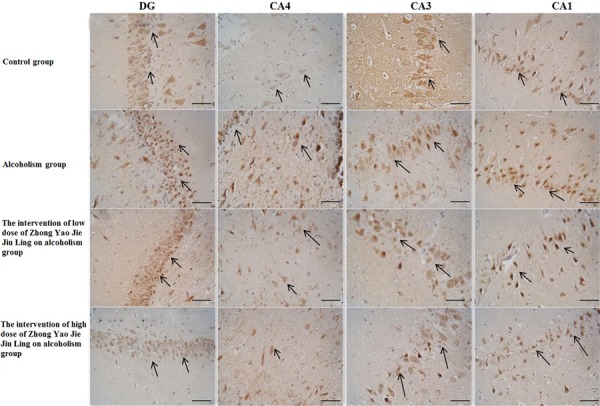
p75 neurotrophin receptor (p75NTR)-positive neurons in the dentate gyrus (DG) and hippocampus (CA4, CA3, and CA1) in the four groups (bars: 100 μm). The arrows indicate p75NTR-positive neurons.

In the hippocampus, p75NTR-positive cells were mainly concentrated in the granular cell layer of the DG and in the pyramidal cell layer of the CA1, CA3 and CA4 regions (Figure 4). The number of positive neurons in these regions decreased in the following order: DG, CA1, CA3 and CA4. Compared with the control group, the number of p75NTR-positive cells in each of these regions was significantly increased in the alcoholism group (P<0.05). Compared with the alcoholism group, the number of p75NTR-positive cells in each of these regions was significantly decreased in the high-dose drug treatment group (P<0.05). In addition, compared with the alcoholism group, the numbers of p75NTR-positive cells in the DG and the CA4 region in the low-dose drug treatment group were also significantly decreased (P<0.05). Furthermore, compared with the alcoholism group, the number of p75NTR-positive cells in the low- and high-dose drug treatment groups were decreased, and there was no statistically significant difference from the control group (P>0.05; Table 3).

**Table:**
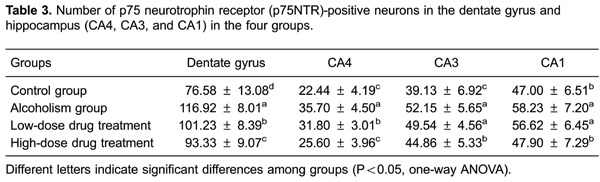

In the control group, pyramidal cells in the CA1, CA3 and CA4 regions were densely arranged, and had long processes and were deeply stained. In the alcoholism group, pyramidal cells in these regions were reduced in number, less well defined and loosely arranged. In the remaining p75NTR-positive neurons, the cytoplasmic staining was noticeably darker, and the nucleus was visible. Granulosa cells in the DG showed similar changes, although changes in their number were more substantial.

## Discussion

In this study, differences in the expression of BDNF and its receptors TrkB and p75NTR in the hippocampus of chronic alcoholic and abstaining dogs were evaluated. BDNF, TrkB and p75NTR-positive cells in the hippocampus were mainly localized in the granular cell layer of the DG and the pyramidal cell layer of the CA1, CA3 and CA4 regions. The number of immunopositive neurons in these four regions decreased in the following order: DG, CA1, CA3 and CA4. In the hippocampus, the expression levels of BDNF and TrkB were reduced in chronic alcoholism, while they were increased in abstinence. In contrast, p75NTR showed an opposite change in expression.

As a chronic disease state, alcohol dependence is associated with neurological illness. Through binding with the TrkB receptor, BDNF activates the MAPK signaling pathway, which plays a major role in alcohol addiction (20). The neurotrophin receptor p75NTR can mediate NGF-induced survival signaling in hippocampal neurons by activating the neurotrophin receptor pathway (27). BDNF is involved in learning and memory by mediating synaptic plasticity and facilitating long-term potentiation (28). BDNF produces axonal morphological changes through TrkB signaling mechanisms that recruit a complex network of signaling pathways. p75NTR signaling pathways can lead to axonal degeneration by suppressing TrkA-mediated signaling (29).

Our findings demonstrated that after long-term heavy drinking, the expression of BDNF decreases. Consistent with this, *in vivo* experiments have shown that acute exposure of neurons to ethanol can lead to increased expression levels of BDNF via the scaffolding protein RACK1, but sustained exposure to ethanol can result in a decrease in BDNF expression (30). In a study of depression, downregulation of BDNF was found in specific hippocampal areas (CA1 and DG), but after antidepressant treatment, BDNF expression significantly increased, indicating that BDNF may play a role in antidepressant therapy (31). This is in accordance with the results of our present study. We found that expression of BDNF and TrkB increased after abstinence, suggesting that abstinence may be essential in protecting the nervous system.

In our study, after nearly 6 months of chronic alcohol intake, BDNF-positive neurons in the hippocampus of chronic alcoholic dogs were significantly fewer than in the control group, similar to its receptor, TrkB. We found that the CA1, CA3, CA4 and DG regions displayed similar changes in expression. Furthermore, it appears that pyramidal cells in the CA4 are more sensitive to alcohol toxicity. However, our findings differ somewhat from a previous study that the expression of BDNF mRNA is increased in the CA2 and DG, but not the CA1 (24), suggesting that different areas of the hippocampus have different sensitivities to alcohol. This contrasting finding might result from differences in the model used and in the abstinence protocol.

In conclusion, changes in BDNF and TrkB expression were positively correlated with each other in the hippocampus in chronic alcoholism and abstinence. In contrast, changes in p75NTR expression were negatively correlated with changes in BDNF and TrkB expression. However, further studies are necessary to unravel the mechanisms underlying the pathogenesis of chronic alcoholism.

## References

[1] Miller MW (2004). Repeated episodic exposure to ethanol affects
neurotrophin content in the forebrain of the mature rat. Exp Neurol.

[2] Santin LJ, Rubio S, Begega A, Arias JL (2000). Effects of chronic alcohol consumption on spatial
reference and working memory tasks. Alcohol.

[3] Samson HH, Harris RA (1992). Neurobiology of alcohol abuse. Trends Pharmacol Sci.

[4] Ryabinin AE (1998). Role of hippocampus in alcohol-induced memory
impairment: implications from behavioral and immediate early gene
studies. Psychopharmacology.

[5] Deitrich RA, Dunwiddie TV, Harris RA, Erwin VG (1989). Mechanism of action of ethanol: initial central nervous
system actions. Pharmacol Rev.

[6] Rosenbaum RS, Winocur G, Moscovitch M (2001). New views on old memories: re-evaluating the role of the
hippocampal complex. Behav Brain Res.

[7] Pang KC, Nocera R, Secor AJ, Yoder RM (2001). GABAergic septohippocampal neurons are not necessary for
spatial memory. Hippocampus.

[8] Walker DW, Barnes DE, Zornetzer SF, Hunter BE, Kubanis P (1980). Neuronal loss in hippocampus induced by prolonged
ethanol consumption in rats. Science.

[9] Walker DW, Hunter BE (1978). Short-term memory impairment following chronic alcohol
consumption in rats. Neuropsychologia.

[10] Rehm J, Shield KD, Gmel G, Rehm MX, Frick U (2013). Modeling the impact of alcohol dependence on mortality
burden and the effect of available treatment interventions in the European
Union. Eur Neuropsychopharmacol.

[11] Birling MC, Price J (1995). Influence of growth factors on neuronal
differentiation. Curr Opin Cell Biol.

[12] Nilsson AS, Fainzilber M, Falck P, Ibanez CF (1998). Neurotrophin-7: a novel member of the neurotrophin
family from the zebrafish. FEBS Lett.

[13] Lai KO, Fu WY, Ip FC, Ip NY (1998). Cloning and expression of a novel neurotrophin, NT-7,
from carp. Mol Cell Neurosci.

[14] Miller R, King MA, Heaton MB, Walker DW (2002). The effects of chronic ethanol consumption on
neurotrophins and their receptors in the rat hippocampus and basal
forebrain. Brain Res.

[15] Kaplan DR, Hempstead BL, Martin-Zanca D, Chao MV, Parada LF (1991). The trk proto-oncogene product: a signal transducing
receptor for nerve growth factor. Science.

[16] Klein R, Jing SQ, Nanduri V, O'Rourke E, Barbacid M (1991). The trk proto-oncogene encodes a receptor for nerve
growth factor. Cell.

[17] Lipsky RH, Marini AM (2007). Brain-derived neurotrophic factor in neuronal survival
and behavior-related plasticity. Ann N Y Acad Sci.

[18] Russo-Neustadt A (2003). Brain-derived neurotrophic factor, behavior, and new
directions for the treatment of mental disorders. Semin Clin Neuropsychiatry.

[19] Davis MI (2008). Ethanol-BDNF interactions: still more questions than
answers. Pharmacol Ther.

[20] Janak PH, Wolf FW, Heberlein U, Pandey SC, Logrip ML, Ron D (2006). BIG news in alcohol addiction: new findings on growth
factor pathways BDNF, insulin, and GDNF. Alcohol Clin Exp Res.

[21] Joe KH, Kim YK, Kim TS, Roh SW, Choi SW, Kim YB (2007). Decreased plasma brain-derived neurotrophic factor
levels in patients with alcohol dependence. Alcohol Clin Exp Res.

[22] Yoon SJ, Roh S, Lee H, Lee JY, Lee BH, Kim YK (2006). Possible role of nerve growth factor in the pathogenesis
of alcohol dependence. Alcohol Clin Exp Res.

[23] Uhl GR, Liu QR, Walther D, Hess J, Naiman D (2001). Polysubstance abuse-vulnerability genes: genome scans
for association, using 1,004 subjects and 1,494 single-nucleotide
polymorphisms. Am J Hum Genet.

[24] Logrip ML, Janak PH, Ron D (2009). Escalating ethanol intake is associated with altered
corticostriatal BDNF expression. J Neurochem.

[25] Wojnar M, Brower KJ, Strobbe S, Ilgen M, Matsumoto H, Nowosad I (2009). Association between Val66Met brain-derived neurotrophic
factor (BDNF) gene polymorphism and post-treatment relapse in alcohol
dependence. Alcohol Clin Exp Res.

[26] He XP, Kotloski R, Nef S, Luikart BW, Parada LF, McNamara JO (2004). Conditional deletion of TrkB but not BDNF prevents
epileptogenesis in the kindling model. Neuron.

[27] Bui NT, Konig HG, Culmsee C, Bauerbach E, Poppe M, Krieglstein J (2002). p75 neurotrophin receptor is required for constitutive
and NGF-induced survival signalling in PC12 cells and rat hippocampal
neurones. J Neurochem.

[28] Lu Y, Christian K, Lu B (2008). BDNF: a key regulator for protein synthesis-dependent
LTP and long-term memory?. Neurobiol Learn Mem.

[29] Singh KK, Park KJ, Hong EJ, Kramer BM, Greenberg ME, Kaplan DR (2008). Developmental axon pruning mediated by
BDNF-p75NTR-dependent axon degeneration. Nat Neurosci.

[30] McGough NN, He DY, Logrip ML, Jeanblanc J, Phamluong K, Luong K (2004). RACK1 and brain-derived neurotrophic factor: a
homeostatic pathway that regulates alcohol addiction. J Neurosci.

[31] Adachi M, Barrot M, Autry AE, Theobald D, Monteggia LM (2008). Selective loss of brain-derived neurotrophic factor in
the dentate gyrus attenuates antidepressant efficacy. Biol Psychiatry.

